# miRNAs as Therapeutic Tools and Biomarkers for Prostate Cancer

**DOI:** 10.3390/pharmaceutics13030380

**Published:** 2021-03-13

**Authors:** Noemi Arrighetti, Giovanni Luca Beretta

**Affiliations:** Molecular Pharmacology Unit, Department of Applied Research and Technological Development, Fondazione IRCCS Istituto Nazionale dei Tumori, 20133 Milan, Italy; noemi.arrighetti@istitutotumori.mi.it

**Keywords:** prostate cancer, miRNA, nanodelivery, metastatic castration-resistant prostate cancer, biomarkers

## Abstract

Prostate cancer (PCa) is the fifth cause of tumor-related deaths in man worldwide. Despite the considerable improvement in the clinical management of PCa, several limitations emerged both in the screening for early diagnosis and in the medical treatment. The use of prostate-specific antigen (PSA)-based screening resulted in patients’ overtreatment and the standard therapy of patients suffering from locally advanced/metastatic tumors (e.g., radical prostatectomy, radiotherapy, and androgen deprivation therapy) showed time-limited efficacy with patients undergoing progression toward the lethal metastatic castration-resistant PCa (mCRPC). Although valuable alternative therapeutic options have been recently proposed (e.g., docetaxel, cabazitaxel, abiraterone, enzalutamide, and sipuleucel-T), mCRPC remains incurable. Based on this background, there is an urgent need to identify new and more accurate prostate-specific biomarkers for PCa diagnosis and prognosis and to develop innovative medical approaches to counteract mCRPC. In this context, microRNA (miRNAs) emerged as potential biomarkers in prostate tissues and biological fluids and appeared to be promising therapeutic targets/tools for cancer therapy. Here we overview the recent literature and summarize the achievements of using miRNAs as biomarkers and therapeutic targets/tools for fighting PCa.

## 1. Introduction

Prostate cancer (PCa) is the third most commonly diagnosed malignancy and the fifth cause of cancer death in men worldwide [1]. Patients suffering from localized/locally advanced disease undergo radical prostatectomy and radiotherapy. Androgen deprivation therapy (ADT) is the treatment of choice for men affected by recurrent or metastatic androgen-sensitive PCa. Unfortunately, ADT fails in several patients who develop the incurable metastatic castration-resistant PCa (mCRPC). Despite the numerous therapeutic options recently proposed, including (i) inhibitors of androgen signaling (enzalutamide) or synthesis (abiraterone); (ii) taxane-based chemotherapy (docetaxel and cabazitaxel); (iii) bone-targeting radiotherapy (radium-223) and (iv) immunotherapy (sipuleucel-T), the medical management of mCRPC still represents an important clinical issue [2]. These pieces of evidence underline the urgent need to develop valuable tools for PCa diagnosis and innovative medical interventions, including new drugs [3,4] as well as drug delivery approaches [5].

Based on the different clinical forms of the disease, ranging from low-grade clinically indolent PCa to aggressive lethal mCRPC, and considering the important side effects of standard therapeutic interventions, it is fundamental to develop tools that allow the identification of patients who require treatments from those not eligible for medical interventions. Patients suffering from low-risk disease (PSA < 10 μg/L, Gleason 3 + 3, clinical-stage ≤ T2a) have a favorable prognosis and can be followed in the context of active surveillance (AS) protocols for avoiding side effects of unnecessary radical prostatectomy or radiation therapy [6]. Currently, PCa diagnosis is based on histopathology test, which is determinant in cases of PSA levels lying in the grey area (4–10 μg/L, normal level 2.5–4 μg/L). Of note, about 25% of patients with PSA levels in the grey area showed positive biopsies. This feature reflects the scarce accuracy of PSA screening tests and warns on diagnosis only based on PSA measurement. However, screening tests are crucial for PCa diagnosis and it has been estimated that their suspension would increase by 13–20% the mortality rate [7]. Based on this background, efforts aimed at developing tests for more precise diagnosis are expected and PCa biomarkers discovery is a booming new wave [8]. In this context, the development of novel tools based on prostate-specific small noncoding single-stranded RNAs (miRNA) showed usefulness in dissecting between BPH and PCa and in defining the disease aggressiveness [9,10]. It is well recognized that up to 30% of the protein-coding genes in the human genome are modulated by endogenously expressed miRNAs. Numerous miRNAs are deregulated in PCa compared to normal cells [11] and altered miRNA profile impacts on gene expression promoting cellular pathways implicated in PCa development and progression [12,13]. Deregulated miRNA profiles may favor oncogenic frameworks that silence tumor-suppressing genes or reduce the activity of tumor suppressor-miRNAs favoring tumorigenesis [9]. In such a way, miRNA-mediated gene expression deregulation impacts tumor development, differentiation, and proliferation, alterations of cell metabolism, apoptosis, senescence as well as immunity response [13,14]. Since miRNAs are released into biological fluids [15] and considering the important progress achieved in miRNA detection in these specimens (e.g., microarrays analysis, RT-PCR, next-generation sequencing, and biosensors), which allow the definition of miRNA expression profiles, these small nucleic acids have become potentially useful biomarkers for the diagnosis and prognosis of cancer, including PCa [16].

Among the numerous approaches considered for fighting cancer, the delivery of nucleic acids, including miRNAs, by nanodevices is very promising. The hypothesis to “repair” deregulated miRNA profiles of cancer cells (e.g., by restoring tumor suppressor-miRNAs or by blocking miRNA oncogene functions) as well as to hit the tumor by administering miRNAs endowed with anticancer properties is intriguing. However, the delivery of naked miRNA suffers from important inconveniences, including the difficulties to pass through plasma membranes and the instability into the biologic fluids [17,18]. In this context, numerous nanovectors for nucleic acid delivery have been proposed [5].

Here we overview the recent achievements of using miRNA as biomarkers and discuss the nanodelivery therapeutic approaches for nucleic acids administration as tools for fighting PCa.

## 2. Prostate Cancer Screening Tests: State of the Art

Alford and co-workers overviewed the several tests available on the market for PCa screening developed for different patient specimens, including (i) Blood: Prostate Health Index (PHI), 4k-score (4-kallikrein panel) and Apifiny; (ii) Urine: SelectMDx, PCA3, Michigan Prostate Score (MiPS); (iii) Biopsy: ConfirmMDx, Oncotype Dx, ProMark, PTEN/TMPRSS2:ERG, Prolaris, Decipher [8]. An ideal screening test is fast and easy to apply to a large population and is based on non-invasive medical practice. Blood and urine specimens are preferred. PHI is based on the detection of truncated proPSA forms, which are abundant in the serum of subjects suffering from PCa. The immunoassay measures the ratio proPSA/freePSA, which is higher in PCa patients. The test, approved by FDA, differentiates benign prostatic hyperplasia (BPH) from suspected PCa cases and showed to improve the diagnosis of high-grade PCa [19,20]. The 4Kscore is based on the measure of four kallikrein blood markers, including intact PSA, free PSA, total PSA, and human kallikrein-related peptide 2. By applying an algorithm that integrates a 4-kallikreins panel, digital rectal examination (DRE), age of the patient, and biopsy, the test predicts high-grade PCa. Although the FDA approval is still lacking, the National Comprehensive Cancer Network recommends 4Kscore for PCa screening [21,22]. Apifiny detects in the blood samples 8 circulating PCa-specific autoantibodies (directed to aurora kinase interacting protein 1, CSNK2A2, NK3 homeobox 1, centrosomal protein 164 kDa, 5ʹ-UTR BMI1, chromosome 3ʹ UTR region Ropporin/RhoEGF, ARF6 and desmocollin 3) in a target population of patients with PSA ≥ 2.5 μg/L [23]. SelectMDx measures the mRNA levels of homeobox C6 (HOXC6, which regulates several oncogenes and tumor suppressor genes implicated in prostate morphogenesis and bone metastasis) and distal-less homeobox 1 (DLX1, which regulates neuroendocrine-epithelial differentiation and predicts PCa aggressiveness) in urine samples after DRE. The test integrates the levels of the two mRNAs with serum PSA, age, and inheritance for PCa. SelectMDx is indicated for men with elevated PSA levels selected for initial prostate biopsy [24]. PCA3 test measures the long noncoding RNA PCA3 level (DD3, found overexpressed in 90% PCa tissues) in the urine samples collected after DRE. Specimens are processed by RT-PCR to define levels of the PCA3 and PSA RNAs. The ratio of the two RNA markers (PCA3 score) predicts PCa. PCA3 is approved by FDA in patients with a prior negative biopsy result [25]. Urine specimens collected after DRE are used for the MiPS test to determine the levels of PCA3 and TMPRSS2:ERG RNAs. TMPRSS2:ERG is a fusion protein that favors PCa invasion found in about 50% of tumors and more frequent in men affected by early-stage disease and low PSA serum levels. The test integrates the two RNA levels with that of PSA in the serum. Men with high PSA levels selected for the first biopsy or with negative first biopsy are enrolled for MiPS [26].

## 3. Overview on Up and Down Regulated miRNAs in Prostate Cancer

Since the discovery of miRNAs and the realization that, compared to normal cells, several miRNAs are aberrantly expressed in tumor cell lines and clinical samples, the study of their functions led to valuable achievements in clarifying the biological processes subtending PCa development and progression [27]. miRNAs are 18–25 nt length noncoding RNA produced in the nucleus. Following the transcription of miRNA genes, the precursor pri-miRNA is processed by RNase III Drosha-DGCR8 complex, resulting in pre-miRNA. Exportin 5 and Ran/GTP61 transport the pre-miRNA into the cytoplasm. The mature double-stranded miRNA is produced by the RNase cleavage activity of RNase III Dicer on pre-miRNA. The targeting of the 3′ UTR of mRNA genes is mediated by the introduction of miRNA into the RNA-induced silencing complex (RISC), which selects the functional strand leading to mRNA degradation, inhibition of the translation, or deadenylation. The non-perfect annealing between miRNAs and target mRNAs implies that miRNAs can act on several targets and in such a way possess many, and sometimes opposite, biological effects. This feature underlines that the identification of the target transcripts, their modulation, and the resulting cellular effects are difficult to study [28,29]. Aimed at managing this issue, numerous website programs have been proposed for the study of miRNAs and their targets. Among these, TargetScan is one of the most accurate and easy to apply, allowing the coupling of miRNAs and mRNAs [30,31]. However, due to the stringent cut-off applied by the program, several targets could be lost. Crucial steps after miRNA target prediction are target validation and functional characterization, which are mandatory to confirm the computational results and to envision the clinical application of a miRNA [28].

Peculiar miRNA and gene expression signatures impact the hallmarks of cancer [32] and have proved efficacy in dissecting between PCa patients and healthy subjects [33,34,35,36]. The usefulness of miRNAs as potential biomarkers relies on their stability in biologic fluids as free nucleic acids or transported by the extracellular vesicles (EVs) [37]. As the demonstration that miRNAs can be detected and measured in biological fluids, miRNA profiles have been proposed as a potential diagnostic and prognostic tool allowing the identification of patients suffering from PCa [38]. miRNAs implicated in cancer development and progression are classified in: (i) onco-miRNAs. These miRNAs are upregulated in cancers and favor carcinogenesis by reducing the levels of tumor suppressor genes; (ii) tumor suppressor-miRNAs. These miRNAs are down-expressed in tumors and promote carcinogenesis by inducing proto-oncogenes expression [39].

Increasing evidence suggest miRNAs as an important class of antisense therapeutic molecules and the correction of altered miRNAs by inhibitors of onco-miRNAs or by mimics of tumor suppressor-miRNAs represents an intriguing medical approach. Numerous miRNAs are under investigation in clinical trials for different diseases, including HCV (miR-122), Alport syndrome (miR-21), nonalcoholic steatohepatitis (miR-103/107), fibrosis (miR-29b), amyotrophic lateral sclerosis (miR-155), inflammatory bowel disease (miR-124), ischemia (miR-92), as well as mesothelioma (miR-16) and lymphoma/leukemia (miR-155) [40]. Based on this background, the identification of miRNAs aberrantly expressed in PCa and the study of the cellular pathways deregulated aroused interest in the development of innovative miRNA-based approaches.

### 3.1. Upregulation of Oncogenic miRNAs

**Figure 1:**
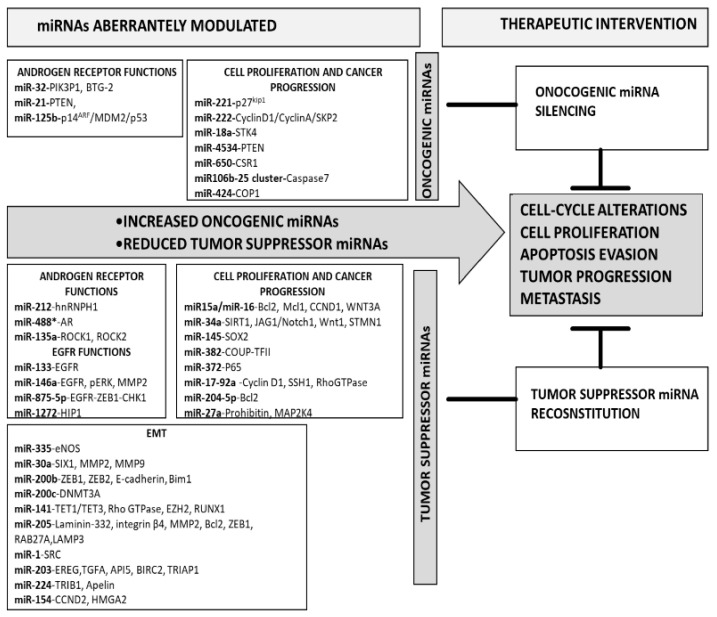
Overview on overexpressed onco-miRNAs and downregulated tumor suppressor-miRNAs in prostate cancer.

Increased expression of miRNAs endowed with oncogenic functions and implicated in cell proliferation, cell-cycle control, and tumor progression is found in PCa, Figure 1 [41,42].

#### 3.1.1. Onco-miRNAs Involved in AR Functions

Among the numerous miRNAs deregulated in PCa, miR-32, miR-21, and miR-125b importantly impact on androgen receptor (AR) expression and functions.

The levels of miR-32 and miR-21 are regulated by AR [43]. Highly expressed in CRPC, miR-32 favors cell growth and proliferation as well as apoptosis evasion by targeting different tumor suppressor genes, including phosphoinositide-3-kinase interacting protein 1 (PIK3IP1) and B-cell translocation gene 2 (BTG2), which inhibit PI3K [44,45]. Increased PI3K activation stimulates AKT/mTOR pathway and promotes cancer development and progression. By silencing PIK3IP1 and BTG2, miR-32 favors PI3K activity and in turn tumor growth. miR-32-mediated downregulation of PIK3IP1 and BTG2 proteins was demonstrated in LNCaP cells, showing low miR-32 levels, transfected with pre-miR-32, and by 3′UTR-luciferase assays. Of note, no BTG2 staining was evidenced in prostatectomy of patients with a short progression-free time.

As miR-32, miR-21 is overexpressed in CRPC and its level correlates with lymph node metastasis, Gleason score, and patient outcome [46]. The transfection of a miR-21 antago-miRNA in PCa cells impacts cell proliferation and invasion by modulating the expression of the tumor suppressor gene PTEN, as well as survivin, Bcl-2, MMP9, MMP2, PI3K/AKT [42,47]. Moreover, the levels of miR-21 measured in prostate biopsies showed to predict recurrence and progression in men suffering from PCa [48,49,50].

Another miRNA involved in AR signaling is miR-125b. AR-mediated enhancement of miR-125b level increases cell proliferation and apoptosis evasion in PCa cell line [51]. By targeting p14^ARF^, miR-125b reduces Mdm2 sequestration/degradation and impairs p53 pathway [52]. Since this behavior is observed in both wild-type (LNCaP) and mutant (22Rv1) p53 cell lines, as well as in p53-null PC3 cells, the oncogene function of miR-125b in PCa occurs through a p53-dependent and -independent mechanism.

#### 3.1.2. Onco-miRNAs Involved in Cancer Progression and Cell Proliferation

A panel of seven onco-miRNAs, including miR-221, miR-222, miR-18a, miR-4534, miR-650, miR-106b-25, and miR-424, controls PCa cell proliferation and progression.

Increased levels of miR-221 and miR-222, which have a very similar seed sequence and are both positioned on the X chromosome, strongly associate with mCRPC progression [53,54]. The two miRNAs reduce the level of p27^kip1^ and in such a way modulate the expression of several genes, including cyclin D1, cyclin A, and S-phase kinase-associated protein 2 (Skp2). This behavior produces increased cell proliferation in vitro and in vivo [53,55,56]. p27^Kip1^ expression is restored in PC3 cells, which show increased miR-221 and miR-222 levels, transfected with antisense LNA oligonucleotides directed against the two miRNAs.

Augmentation of miR-18a levels paralleled with increased cell proliferation and apoptosis evasion mediated by the downregulation of the tumor suppressor serine/threonine-protein kinase 4 (STK4) and elevated levels of Akt phosphorylation. This behavior is counteracted by the miR-18a inhibition both in vitro and in vivo [56].

Compared to normal prostate cells, reduced methylation of miR-4534 is observed in PCa. This feature increases miR-4534 expression that in turn reduces PTEN levels [57]. The overexpression of miR-4534 in normal RWPE1 prostate cells favors a tumor-prone phenotype [58]. Conversely, the silencing of miRNA in DU145 and MDAPCa2b cell lines induces apoptosis, cell cycle, and growth arrest [58]. This finding was confirmed in vivo in the DU145 xenograft mouse model.

The oncogenic behavior of miR-650 was demonstrated in PCa by Zuo and colleagues [59]. Increased levels of miR-650 impact on PCa tumorigenesis by reducing the expression of the cellular stress response 1 (CSR1) protein. The silencing of miR-650 in DU145 and PC3 cells restores the expression of CSR1, arrests the cell cycle and cell proliferation.

Similarly, upregulation of the miR-106b-25 cluster is observed in primary PCa tumors and distant metastasis and associates with low caspase 7 expression and CRPC progression. By silencing caspase7, the cluster favors tumor progression and spread [54]. In vitro studies in LNCaP and DU145 cell lines and 3′-UTR luciferase assay demonstrated that caspase7 is the direct target of miR-106b. Additionally, the lentiviral overexpression of the cluster in 22Rv1 cells results in enhanced adhesion to the basement membrane and increased soft agar growth. This feature argues for the involvement of miR-106b in cell focal adhesion pathways.

As far as miR-424 is concerned, the existence of a miR-424/COP1/STAT3 axis was reported by Dallavalle and colleagues [60]. miR-424 targets the E3 ubiquitin ligase COP1 and induces STAT3 activity. Normal RWPE1 prostate epithelial cells supplemented with a miR-424 switch toward a tumor-prone phenotype. Conversely, anti-miR-424 transfection in DU145 and LNCaP cells reversed the tumor phenotype both in vivo and in vitro.

### 3.2. Downregulation of Tumor Suppressor-miRNAs

Downregulation of tumor suppressor-miRNAs is observed in PCa (Figure 1), and such a behavior enhances cell proliferation, epithelial–mesenchymal transition (EMT), invasion, and metastasis.

#### 3.2.1. Tumor Suppressor-miRNAs Involved in AR Functions

The loss of miRNAs controlling AR expression parallels with PCa promotion and progression. Altered expression of miR-212, miR-135a, and miR-488* modulates AR level impacting on PCa development and progression [61,62,63]. Normal levels of miR-212 downregulate hnRNPH1 expression that reduces AR amount. The loss of miR-212 upregulates AR via increased expression of hnRNPH1 [61]. This finding is corroborated by the reduced expression of hnRNPH1 transcripts, as well as AR and AR-V7 variants, observed after ectopic expression of miR-212 in the C4-2B PCa cell line.

The expression and functions of AR are regulated by miR-488*, as well. High miR-488* levels inhibit proliferation and favor apoptosis via downregulation of AR in PCa cell lines, including LNCaP, C4-2b, and DU145 [63].

Another miRNA under the control of AR signaling is miR-135a. Increased transcription of miR-135a is observed following androgen stimulation in PC3 cells. By downmodulating ROCK1 and ROCK2, miR-135a reduces PCa migration and invasion in vitro and in vivo, as shown in the xenograft mouse model of miR-135a-overexpressing PC3 cells [62].

#### 3.2.2. Tumor Suppressor-miRNAs Involved in EGFR Functions

EGFR signaling controls PCa aggressiveness and progression. Reduced expression of miR-133, miR-146a, and miR-875-5p increases EGFR signaling and favors PCa progression and angiogenesis [64,65,66].

Very low levels of miR-133 are observed in PC3 and DU145 cells and the ectopic supplementation of miR-133 reduces EGFR expression, cell proliferation, and migration/invasion capability.

EGFR signaling is also modulated by miR-146a. Xu and colleagues demonstrated that, besides EGFR levels, miR-146a reduces the expression of p-ERK and MMP2, which are involved in PCa cell proliferation and invasion, as observed in DU145 and PC3 cell lines. This finding was confirmed in vivo in mice bearing miR-146a-overexpressing DU145 tumor xenografts [64].

The EGFR direct targeting of miR-875-5p was demonstrated by El Bezawy and coworkers in DU145 and PC3 cell lines [65]. The exogenous administration of miR-875-5p reduces EMT and enhances radiation-induced DNA damage by impairing EGFR nuclear translocation and ZEB1/CHK1 axis.

Another miRNA with tumor-suppressive functions that act on EGFR membrane turnover and downstream AKT/ERK pathway is miR-1272 [67]. The transfection of miRNA mimic in DU145 and 22Rv1 cells reduces cell migration and invasion and reverses EMT by targeting HIP1, which in turn alters the membrane distribution of EGFR. Of note, miR-1272 rescue improves ionizing radiation sensitivity in vitro and in vivo.

#### 3.2.3. Tumor Suppressor-miRNAs Involved in EMT

Typical biochemical and cellular changes occur in cells undergoing EMT, including the acquisition of mesenchymal features and the loss of epithelial properties, the reduction of the cell to cell attachment, as well as the modulation of extracellular matrix [68]. Several miRNAs modulate the expression of genes involved in EMT and their loss/reduced expression favors tumor invasion, metastasis spread, and influences drug response.

The reduced expression of miR-335 is found in PCa tissues and cell lines, including PC3, LNCaP, and DU145 [69]. Endothelial nitric oxide synthase (eNOS) is the target of miR-335 and the ectopic expression of the miRNA in PC3 cells reduces the eNOS level and counteracts cell migration and invasion in vitro.

Reduced levels of miR-30a are observed in PCa cells, including C4-2, 22Rv1, DU145, and PC3, which show increased proliferation and invasive phenotype. Zhu and coworkers demonstrated that the reduced level of miR-30a correlated with increased expression of sine oculis homeobox homolog 1 (SIX1), MMP2, and MMP9, and that this feature promotes PCa cell growth and invasion [70]. Of note, this behavior was reversed when the level of miR-30a was restored in PC3 and DU145 cells.

Another miRNA crucial for EMT is miR-200b [71]. By targeting the zinc-finger E-box binding homeobox 1 and 2 factors (ZEB1 and ZEB2), E-cadherin and Bim1, miR-200b maintains the epithelial phenotype and suppresses cell proliferation, EMT, and tumor invasiveness [72]. The exogenous administration of miR-200b in PC3 cells downregulates ZEB1 and ZEB2 and reverses the EMT phenotype.

Beside miR-200b, also miR-200c regulates EMT [73,74]. Banyard and colleagues selected metastatic cancer cells from the lymph nodes of mice bearing orthotopic DU145 tumors. The metastatic cells showed a mesenchymal-epithelial transition (MET) phenotype, e.g., increased levels of epithelial markers (cytokeratin 18, E-cadherin, EpCAM) and low levels of the mesenchymal marker vimentin. Importantly, the MET phenotype correlated with increased expression of miR-200c, and the administration of selective anti-miR-200c reversed MET toward EMT phenotype. Prostate biopsies show that the levels of miR-200c and miR-141 inversely correlate with methylation in CpG sites of promoter closest to the miRNA sequence and in vitro supplementation of miR-200c and miR-141 in PC3 cells reduces the levels of DNA methyltransferase 3 alpha (DNMT3A) or TET1/TET3, respectively. These features imply the existence of miR-200c-DNMT3A and miR-141-TET1/TET3 cross-talks. Moreover, reduced cell growth and apoptosis induction are observed in miR-200c- or miR-141-restored PC3 cells [75]. This behavior is cell line dependent and, conversely to PC3 cells, LNCaP, DU145, and 22RV1 cells showed unmethylated promoter and normal levels of miR-200c and miR-141. Liu et al. observed a reduced expression of miR-141 in PCa patient samples [76]. Forced expression of miR-141 in PC3 and DU145 cell lines provokes the switch from EMT to MET phenotype, reduces cell proliferation, and suppresses tumor regeneration in vivo. The targets modulated by miR-141, including Rho GTPase family members (CDC42, CDC42EP3, RAC1, and ARPC5) and EZH2 have been validated. An inverse correlation between the reduced level of miR-141 and runt-related transcription factor 1 (RUNX1) expression in patient specimens was reported by Xu and colleagues [77]. Exogenous supplementation of miR-141 in PC3 and DU145 cell lines reduces MMP9, MMP2, and RUNX1 and enhances FOXO1 and p21 expression. This finding parallels with reduced cell growth, migration, and invasion and with increased apoptosis. However, the functions of miR-141 in PCa appear controversial. Its expression is found increased after castration arguing for an implication in androgen-related mechanisms [78]. This statement is corroborated by Khorasani et al. that reported an inverse correlation between high miR-141 levels and the expression of the AR co-repressor protein small heterodimer partner gene (SHP/NR0B2) in metastatic PCa samples [79].

Another miRNA implicated in EMT is miR-205. Numerous targets under the control of this miRNA, including Bcl2, laminin-332, integrin-β4, and MMP2, are involved in cell changes toward EMT phenotype. Increased cell proliferation and invasiveness, as well as resistance to apoptosis, are observed in PCa with miR-205 downregulation [80,81]. The host gene of miR-205, MIR205HG, transcripts a nuclear long noncoding RNA that maintains the identity of prostate epithelial cells [82]. Gandellini and colleagues demonstrated that ΔNp63α controls miR-205 expression that in turn regulates the deposition of laminin-332 and the levels of integrin-β4. This behavior is critical for cell physiology and the loss of miR-205 generates basement membrane discontinuity that favors tumor invasion [80]. miR-205 levels play a pivotal role in chemotherapy response, as well. The ectopic supplementation of miR-205 in DU145 and PC3 cells enhances radiation response and impairs autophagy in vitro and in vivo [83,84,85]. miR-205-reconstituted PCa cells show downregulation of PKCε that impairs EGFR nuclear translocation and DNA-PK activation resulting in a radiosensitizing effect [84]. Additionally, miR-205 supplementation in PCa cells reduces the levels of RAB27A and LAMP3 altering the tumor autophagic milieu and restoring cell sensitivity to antitumor drugs [85]. Another target of miR-205 is the antiapoptotic protein Bcl2. The miR-205-mediated downregulation of Bcl2 was demonstrated in PCa cell lines by Verdoodt and coworkers [81]. Compared to scramble-transfected cells, miR-205-transfected PC3 and LNCaP cells showed increased apoptosis induction following exposure to DNA damaging drugs. Of note, Bcl2 mRNA expression in PCa biopsies correlated with extracapsular extension of the tumor.

By targeting transforming growth factor-alpha (TGF-α), tribbles pseudokinase 1 (TRIB1), CCND2, and high-mobility group AT-hook 2 (HMGA2) and Src, miR-203, miR-224, miR-154, and miR-1, respectively, counteract EMT, tumor progression and metastasis. Their loss/downregulation associates with PCa promotion and dissemination [86,87,88].

An AR-miR-1-SRC network was proposed for PCa progression and bone metastasis by Liu et al. [86]. Since the increased expression of SRC (a proto-oncogene tyrosine-protein kinase that favors cell survival, proliferation, invasion as well as angiogenesis) in clinical samples of CRPC patients associated with reduced levels of miR-1 and with low AR gene signature expression, AR was proposed to regulate miR-1 transcription and in turn SRC levels. In this scenario, the loss of androgen-regulated miR-1 activates SRC and promotes PCa bone metastasis. This finding is corroborated by the exogenous introduction of miR-1 in DU145 cells, which showed ERK signaling inhibition and attenuated cell invasion, as well as reduced bone metastasis in vivo.

A molecular link between miR-203 and EGFR-dependent gene signature was observed in PCa patients suffering from bone metastasis [87]. A reduced miR-203 expression correlates with increased levels of EREG and TGFA (two EGFR ligands) and with the augmentation of the anti-apoptotic API5, BIRC2, and TRIAP1 proteins. This behavior reflects the resistance to EGFR inhibitors (CI1033 and AG1478) observed. The transfection of miR-203 in DU145 cells restored sensitivity to tyrosine kinase inhibitors and attenuated bone metastasis spread in vivo.

Another miRNA deregulated in PCa compared to normal tissues is miR-224. The reduced expression of this miRNA associates with increased TRIB1 and PSA levels, as well as with metastasis and poor prognosis. DU145 cells transfected with miR-224 mimic showed increased apoptosis via downmodulation of TRIB1 and reduced cell proliferation, migration, and invasion [88]. Another target of miR-224 is apelin (APLN). By targeting APLN, miR-224 inhibits LNCaP cell invasion and migration [89]. The ectopic administration of miR-224 in PC3 and DU145 cells arrests cell-cycle and impairs cell adhesion and locomotion. Advanced clinical stage and metastasis in PCa patients correlated with miR-224 reduced expression in biopsies [90].

miR-154 is also downregulated in PCa compared with normal tissues. Forced expression of miR-154 in PC3 and DU145 cells decreases the levels of CCND2 and high-mobility group AT-hook 2 (HMGA2) and in such a way reduces cell growth, cell locomotion, and EMT [91,92].

#### 3.2.4. Tumor Suppressor-miRNAs Involved in Cancer Progression and Cell Proliferation

Several miRNAs with tumor suppressor properties target proteins implicated in controlling cell proliferation, cell-cycle as well as apoptosis.

PCa mouse models revealed that the loss of miR-15 and miR-16 and the concomitant overexpression of miR-21 promote cancer progression. By reducing the levels of Bcl2, Mcl1, CCND1, and WNT3A, miR-15a and miR-16 exert tumor suppressor functions [93,94]. The administration in vivo of antago-miRNA directed against miR-15a and miR-16 produces prostate hyperplasia and favors a tumor-prone phenotype. Conversely, tumor regression, growth arrest, and apoptosis induction are observed in miR-15a- and miR-16-reconstituted LNCaP xenograft tumors. Of note, patients suffering from PCa show miR-15/miR-16 and miR-21 aberrant deregulation that reflects the clinical outcome [95].

Another tumor suppressor-miRNA modulated in PCa is miR-34a. PC3 cells resistant to paclitaxel showed reduced expression of miR-34a that paralleled with SIRT1, HuR, and Bcl2 overexpression. Exogenous supplementation of miR-34a precursor counteracts paclitaxel resistance by direct targeting to the 3′-UTR of SIRT1 mRNA. The augmentation of the miRNA reduces the expression of HuR and Bcl2, as well [96]. By using luciferase reporter assay, Liu and colleagues demonstrated that miR-34a directly targets also JAG1 and Notch1. The supplementation of miR-34a in PC3 cells resistant to paclitaxel restores the JAG1 and Notch1 pathways and the sensitivity to the antitumor drug [97]. Dong and coworkers reported the involvement of miR-34a in modulating the Wnt/β-catenin pathway by targeting the Wnt1 gene. The reconstitution of miR-34a in PC3 cells arrests the cell-cycle progression and reduces EMT, cell proliferation, and migration, and favors apoptosis [98]. Another direct target of miR-34a is the oncogenic factor Stathmin-1 Oncoprotein (STMN1) that controls the expression of growth differentiation factor 15 (GDF15). By reducing the expression of STNM1, the ectopic supplementation of miR-34a in PC3 and DU145 cells reduces proliferation and colony formation [99]. Among the strategies considered for the treatment of PCa, the exogenous supplementation of miR-34a proved efficacy [100].

miR-145 was also reported to possess tumor-suppressive functions in PCa. The reduced expression of miR-145 in PCa biopsis correlated with high Gleason score, clinical stage, high PSA levels, tumor size, and a higher risk of disease recurrence [101]. This finding parallels with the reduced levels of miR-145 observed in PC3, DU145, and LNCaP cell lines. The overexpression of miR-145 in PCa cell lines caused reduced proliferation and migration via reduction of SOX2 expression. Conversely, its inhibition in normal PNT1a cells favors transformations toward tumorigenesis [102].

Normal levels of miR-382 are responsible for reduced expression of the chicken ovalbumin upstream promoter transcription factor II (COUP-TFII), Snail, and MMP2, which reduce cell proliferation and invasion. The restoration of the COUP-TFII levels by miR-382 ectopic supplementation in LNCaP and PC3 cell lines counteracts cell proliferation and invasion [103].

Another miRNA implicated in cell proliferation and invasion is miR-372. DU145 cells, which show low levels of this miRNA, supplemented with miR-372 reduce cell proliferation and migration. The validated target of miR-372 is p65, which acts on cell proliferation and migration by controlling CDK8 and MMP9 [104].

Six mature miRNAs, including miR-17, miR-18a, miR-19a, miR-19b, miR-20a, and miR-92a, are the transcripts of the miR-17-92a cluster. The cluster is downexpressed in PCa specimens and LNCaP and PC3 cell lines. Ectopic administration of the cluster in PCa cell lines downregulates cyclin D1, SSH1, and proteins of RhoGTPase and MAP kinases pathways. This behavior in turn reduces cell proliferation, EMT, and tumor growth in vivo. Importantly, the restoration of the cluster positively impacts the sensitivity to chemotherapy [105].

The expression of miR-204-5p is reduced in PCa cell lines, including PC3, 22Rv1, LNCaP, in comparison to normal prostate epithelial cells. The antiapoptotic protein Bcl2 is the direct target of miR-204-5p. miRNA rescue in PCa cell lines reduces Bcl2 levels favoring cytochrome C release as well as caspase 3/7 activation and apoptosis induction [106].

miR-27a is also a tumor suppressor found downregulated in CRPC cells, including LNCaP. By targeting prohibitin and MAP2K4, miR-27a is crucial for cell proliferation, cell-cycle control, and apoptosis. The rescue of miR-27a levels in LNCaP cells reduces cell proliferation and migration [107,108].

Let-7 gene transcripts a highly conserved miRNA family and in PCa tissue, the loss of let-7 family correlated with increased expression of EZH2 and paralleled with higher Gleason score and tumor stage [109]. By targeting different oncogenes, among which, RAS, Lin28, HMGA2, and c-Myc, the miRNAs belonging to the Let-7 family control EMT, cell-cycle, cell proliferation, differentiation, and migration [110,111,112].

## 4. miRNA Delivery Approaches for Prostate Cancer Treatment

The implication of specific miRNAs in several cellular pathways involved in PCa development and progression, together with the possibility to modify their cellular expression levels, opened an interesting scenario for the medical management of this disease [113,114,115,116,117,118]. The approaches for miRNA-based therapy include the silencing of onco-miRNAs or the reconstitution of tumor suppressor-miRNAs (Figure 1 and Table 1).

The inhibition of onco-miRNAs is achieved by (i) antisense oligonucleotides (ASOs, also called antago-miRNAs or anti-miRNAs). ASOs, which include cholesterol-conjugated and locked nucleic acid (LNA) anti-miRNAs, are single-stranded RNAs that interact with miRNAs impeding their binding with mRNAs; (ii) miRNA sponges, which are regulatory RNAs containing multiple tandem binding sites capable of interacting with numerous miRNA targets and dramatically depleting miRNAs cellular content; (iii) small molecules, which recognize peculiar structures of a specific miRNA and interfere with its biogenesis or maturation and iv) CRISPR/Cas9 genome editing. A new genetic approach is based on the editing of the miRNA-containing gene to suppress miRNA expression [119]. Conversely, the restoration of tumor suppressor-miRNAs, miRNA replacement therapy, includes the administration of (i) miRNA mimics (e.g., ago-miRNAs); (ii) viral vectors encoding for miRNAs and (iii) small molecules that improve the endogenous expression of miRNAs by epigenetic changes within the gene structure [120].

Although the RNA-based therapies provide the advantage of enhanced specificity compared to conventional chemotherapeutics, the instability and poor bioavailability of miRNAs limit their clinical applications [121]. Small RNAs suffer from serum nuclease-mediated degradation and rapid clearance by renal excretion. Additionally, systemic delivery of nucleic acids activates the immune system surveillance inducing immunotoxicity [122]. To overcome these limitations, chemical modifications improving RNA’s stability and reducing side effects have been proposed. In this context, ago-miRNA containing cholesterol and 4 thiol moieties at the 3′ terminal, 2 thiol groups in the 5′ terminal of a 2’-methoxy-modified full-length nucleotide proved increased stability and affinity for cell membrane, compared to unmodified miRNA [123]. The tumor suppressor miR-133a-3p was restored in a mouse model bearing PC3 tumors by ago-miRNA intravenous injection. By inactivating PI3K/AKT pathway and targeting multiple cytokine receptors (i.e., EGFR, FGFR1, IGFR1, and MET), the ago-miRNA efficiently reduced tumor spread and bone metastasis [124].

Chemical modifications of nucleic acids partially overcome nuclease degradation and immune system activation [125]. Conversely, miRNA-based therapeutics scarcely penetrate cell membranes and, due to their small size, are susceptible to rapid renal clearance [126,127]. To overcome these inconveniences, different nanoparticles, including lipid-, cationic polymer- and peptide-based vectors endowed with high nucleic acid loading capacity have been proposed [128]. Cationic polymers have good biocompatibility, low toxicity, and, due to electrostatic features, high transfection efficacy [129]. Among the cationic polymers, polyethyleneimine (PEI) showed good transfection capacity, cargo protection from enzymatic degradation, as well as endosome escaping through the “proton sponge effect” [130]. PEI has been used to replace the tumor suppressor miR-413 in a PC3 xenograft mouse model. A significant tumor growth inhibition was observed in mice treated with PEI-miR-143. Radiolabeled miRNA-PEI complexes evidenced a strong miRNA accumulation in tumor, lung, and spleen, confirming the usefulness of PEI as a miRNA delivery system. However, PEI nanoparticles are cytotoxic and aggregate following blood injection [131,132]. These drawbacks were overcome by conjugating PEI with other molecules (i.e., cholesterol, polyethylene glycol-PEG) or other nanoparticles [133,134]. Nagesh and colleagues developed PEI-PEG shell nanoparticles containing an iron oxide magnetic core that efficiently delivered the tumor suppressor miR-205 to C4-2 and PC3 cells. These nanoparticles escaped the endolysosomal pathway and released intact miR-205 into cytoplasmic compartments. In vivo experiments demonstrating the improvement of the toxicity profile, due to PEG modification, are not available, yet [135]. PEI functionalized polyhydroxybutyrate nanoparticles (PHB-PEI) were used to deliver miR-124, a modulator of carnitine palmitoyltransferase 1A (CPT1A), a key enzyme of mitochondrial fatty acid oxidation. PHB-PEI-miR-124 complexes were used to treat PC3 cells and showed high cellular uptake and decreased CPT1A expression, in turn leading to reduced cell proliferation and motility [136]. Extracellular vesicles (EVs) conjugated with PEI proved efficacy in delivering nucleic acids [137]. EVs isolated from PC3 cells were conjugated with PEI-anti-miR-155, which high levels are reported to restore PTEN and TP53INP1 expression. PC3 cells treated with EVs-PEI-anti-miR-155 increased PTEN and TP53INP1 expression and showed reduced proliferation [138]. To enhance endo/lysosomal escape, a PEI-modified nanovector (PMPC) made of PEI-(4-(bromomethyl) phenylboronic acid) (PBA) and PEI-(2,3-dimethylmaleicanhydride) (DMA) was engineered. These nanoparticles were used to co-deliver miR-146a and cetuximab, both targeting EGFR, in PCa cells. PMPC nanoparticles contain an inner core polyplex (PEI−PBA-miR-146a) bound to the outer layer polyplex (PEI−DMA-cetuximab) via electrostatic interactions. PMPC showed biocompatibility and cargo protection from enzymatic degradation. In vitro experiments performed in DU145 cells confirmed the miR-146a-mediated EGFR silencing, reduced cell growth, invasion, and migration. PMPC-loaded nanocomplexes strongly inhibited DU145 tumor growth in vivo [139].

Another polymer proposed for nucleic acid delivery is hyperbranched poly(amido amine) (HPAA). HPAA-nanovectors escaped endolysososml pathway and showed rapid cargo release [140]. Specific targeting of PCa cells was achieved by engineering a PEG-modified HPAA conjugated with an aptamer recognizing the prostate-specific membrane antigen (HPAA-PEG-APT). LNCap cells treated with miR-133-3p-loaded HPAA-PEG-APT showed reduced cell viability and decreased expression of miR-133-3p target proteins, including MET and EGFR. The antitumor potency of HPAA-PEG-APT/miR-133a-3p in vivo was demonstrated in mice bearing LNCap xenograft tumors [140].

Ultrasound-targeted microbubble destruction (UTMD) is a novel non-invasive system for nucleic acid delivery. Microbubbles are systemically injected and destroyed by topical ultrasounds application on the target site [141]. PC3 cells exposed to combined miR-205-loaded UTMD and ultrasounds strongly reduced proliferation and motility and showed increased sensitivity to cisplatin [142].

Redox-sensitive amphiphilic micelles (VPs) are composed of a hydrophilic shell containing disulfide bridges linked to low molecular weight protamine encapsulating a hydrophobic core of Vitamin E succinate. This chemical structure favors cell penetration, endosomal escape, and rapid cargo release into the cytoplasm due to the reduction of disulfide bonds mediated by red-ox features of the cytosol, which promotes micelles destruction. Docetaxel/miR-4638-5p-loaded VPs showed efficient tumor-targeting ability followed by a sustained miR-4638-5p and docetaxel release both in vitro and in vivo in PCa models. Enhanced apoptosis and cell-cycle arrest were observed in PC3 and DU145 cells exposed to VPs-loaded micelles. In mice bearing PC3 tumors, VPs accumulated into the tumor and liver and significantly reduced tumor growth compared to free docetaxel. The decreased expression of Kidins220 and p-AKT, two targets of miR-4638-5p, confirmed the capability of VPs to deliver miRNAs [143,144].

Currently, few clinical trials containing miRNAs are ongoing in cancer. No study enrolls PCa patients.

## 5. The Diagnostic and Prognostic Power of miRNAs in Prostate Cancer

Since the demonstration in 2008 that miRNAs released into the blood circulation of mice bearing 22Rv1 xenograft tumors are very stable, also after several cycles of freeze and thaw, can be easily measured by PCR-based techniques and, more importantly, can separate mice bearing tumors from controls, the analysis of the miRNA profiles of patients suffering from cancer, including PCa, attracted attention [38]. Indeed, in the same study, Michell and coworkers also demonstrated that the serum levels of miR-141 robustly separated metastatic PCa patients from healthy subjects. Besides blood, miRNAs were hereafter identified in numerous biologic fluids, packaged with EVs, assembled with nucleophosmin or argonaute 2 proteins, as well as associated with high-density lipoproteins [22]. The studies focused on circulating miRNAs are conflictual, some of them report that released miRNAs are mostly associated with argonaute 2 and others that miRNAs are preferentially packaged into the EVs [15,145,146,147,148]. Moreover, platelet contamination or hemolyzed samples are responsible for technical bias that negatively impacts miRNA profile [149,150,151].

The analysis of circulating miRNAs in patients suffering from PCa, matched together with the clinical variables, has shown potential usefulness in disease diagnosis and prognosis, Table 2.

### 5.1. Diagnostic miRNA Profiles

miR-1246 was reported by Bhagirath and colleagues as a potential diagnostic PCa biomarker predicting disease aggressiveness [152]. Patients suffering from aggressive PCa or BPH and control subjects were recruited and serum samples analyzed for miRNA levels. Among the several deregulated miRNAs, the reduced levels of miR-1246 significantly distinguished the 3 populations and correlated with disease grade, metastasis, and poor prognosis.

The diagnostic power of miR-150-5p in liquid biopsy was proposed by Paunescu and coworkers [153]. The study collected paired biopsy and plasma of patients affected by PCa and following the comparison of the miRNA profiles, 5 modulated miRNAs, including miR-130a-3p, miR-145-5p, miR-148a-3p, miR-150-5p, and miR-365a-3p, were found. Among these, miR-150-5p was robustly downregulated in both tissues (AUC 0.809, 95% CI: 0.616–1.001) and plasma (AUC 0.817, 95% CI: 0.680–0.995) evidencing the concordance between the two types of specimens and its usefulness for PCa diagnosis.

In another study, the serum levels of miR-141, miR-21, and miR-375 proved efficacy in PCa diagnosis. The study enrolled men (*n* = 20) affected by PCa with a PSA mean of 21.3 µg/L and healthy controls (8 subjects). Compared to controls, increased levels of the 3 miRNAs were observed in PCa patients. Among the miRNAs found increased, the levels of miR-375 showed the best ROC performance (AUC 0.906, 95% CI: 0.797–1.001) [154].

A combination of 4 biomarkers, including PCA3, miR-141, kallikrein 2, and PSA were evaluated in 100 subjects stratified in PCa and BPH patients according to clinical diagnosis. The study demonstrated that the combination of PCA3, miR-141, and kallikrein 2 separated BPH from PCa subjects and showed interesting performance in identifying PCa in patients with PSA levels lying in the grey area (4–10 µg/L) [155].

The levels of miR-98-5p, miR-152-3p, miR-326, and miR-4289 were measured in plasma samples of PCa patients and healthy controls and considered for PCa diagnosis. The comparison of the signatures showed high specificity and sensitivity in detecting patients with PCa (AUC 0.88). The miRNAs were significantly upregulated in PCa patients compared to controls and, since based on existing literature and data the panel was not found in tumors of different origin, Moya and colleagues speculated that the signature represents a specific diagnostic tool for PCa [156]. The same panel reported by Moya and colleagues was published in the study by Matin et al. [157]. Plasma samples from patients affected by PCa before, during, and after treatment, and from healthy subjects were collected and miRNA levels evaluated. Significantly increased expression of the 4 miRNAs was detected in PCa compared to healthy individuals (AUC 0.88, 95% CI = 0.82–0.94, *p* < 0.0001). The interrogation of the published miRNA transcriptomic data for the 4 miRNAs in PCa and corresponding normal adjacent tissue biopsies showed concordance for miR-152-3p expression.

A panel of 5 parameters including PSA, prostate volume, and serum levels of miR-4286, miR-27a-3p, and miR29b-3p was considered by Lyu et al. [158]. The panel was compared in PCa (*n* = 78) and BPH (*n* = 77) patients and revealed excellent diagnostic performance in separating the two populations (AUC 0.892, 95% CI: 0.832–0.937, sensitivity 78.95%, and specificity 92.21%) as well as in recognizing the early-stage disease. The results were validated and confirmed in a cohort of serum samples from 100 individuals.

PCa and BPH patients were recruited in the study by Worst and colleagues for the definition of the diagnostic power of 3 miRNAs panel, including miR-10a-5p, miR-99b-5p, and miR-29b-3p [159]. The study collected tissues and plasma samples from patients suffering from PCa (*n* = 18) and BPH (*n* = 7) and the levels of miRNAs were measured. miR-10a-5p and miR-29b-3p were statistically incremented in plasma of PCa compared to BPH. No different expression was evidenced in tissue biopsies.

In another study, Ibhaim et al. proposed miR-21, miR141, miR18a, and miR-221 levels for PCa diagnosis [160]. Plasma samples from PCa (*n* = 50 localized, *n* = 30 metastatic), BPH (*n* = 30) and controls (*n* = 50) were collected and miRNA levels measured. Among the miRNAs found differentially expressed, miR-18a significantly separated PCa from controls (AUC 0.966, 95% CI, 0.937–1.000) and the combination miR-18a and miR-221 robustly segregated localized from metastatic PCa (AUC 0.997, 95% CI, 0.988–1.0).

Cai and colleagues showed a correlation between miR-494 serum levels and tumor stage [161]. The serum levels of miR-494 were analyzed in PCa (*n* = 90) and BPH (*n* = 90) patients as well as in healthy voluntaries (*n* = 90). The expression of miR-494 was increased in BPH compared to control and was significantly higher in PCa compared to BPH patients (AUC 0.8090, 95% CI, 0.7343–0.8837). Besides separating the 3 populations, miRNA levels correlated with Gleason score, tumor stage, and PSA levels of PCa patients.

Mello-Grand and colleagues proposed the combination of plasma levels of miR-103a-3p, let-7a-5p, and PSA for PCa diagnosis [162]. The study measured the levels of the 2 miRNAs and PSA in samples from PCa (*n* = 60) patients and controls (*n* = 60) and matched the results with patient clinical variables by applying an empirical Bayesian approach and multivariate penalized logistic regression. The diagnostic model was applied in a validation set and showed to detect PCa significantly better than PSA alone.

The diagnostic potential of miR-324 was reported by Jin and colleagues [163]. The study collected patients affected by PCa (*n* = 50), BPH (*n* = 30) and control (*n* = 20) individuals and the serum levels of miR-324 evaluated. Significantly increased levels of miR-324 were observed in PCa compared to BPH and control subjects (AUC 0.911, 95% CI: 0.855–0.966), and miRNA expression positively correlated with PSA levels, with Gleason score and tumor stage.

Another diagnostic miRNA panel was recently proposed by Fredsøe and colleagues [164]. The study profiled plasma samples (*n* = 753) of patients affected by PCa in different stages, including BPH, localized and advanced disease, as well as control subjects. The analysis resulted in a miRNA ratio model (miR-375-miR-33a-5p/miR-16-5p-miR-409-3p) that in combination with the age of patients, PSA levels, and DRE robustly predicted the outcomes of transrectal ultrasound-guided biopsies with superior accuracy than PSA alone (AUC: model 0.67, PSA alone 0.56).

PCa diagnosis based on the plasma levels of miR-141-3p and miR-125a-5p was proposed by Li et al. [165]. Plasma samples from PCa (*n* = 31) patients and control (*n* = 19) individuals were collected and analyzed for miRNA expression. Compared to healthy controls, PCa patients showed increased miR-141-5p (AUC 0.652) and reduced miR-125a-5p (AUC 0.691) levels and the ratio miR-125a-5p/miR-141-5p robustly separated PCa patients from healthy subjects (AUC 0.793).

Increased levels of miR-374b-5p in peripheral blood were proposed as a potential biomarker by Pang et al. [166]. The study recruited samples from PCa (*n* = 42) and BPH (*n* = 42) patients, as well as healthy (*n* = 42) controls and the levels of miR-374b-5p measured. Elevated miR-374b-5p expression, which paralleled with Gleason score and PSA levels, was observed in PCa patients compared to BPH and controls. ROC analysis showed the power of the miRNA in distinguishing PCa from control individuals (AUC 0.851, 95% CI, 0.766–0.936) and PCa from BPH patients (AUC 0.831, 95% CI, 0.742–0.920).

The serum levels of 10 miRNAs, including miR-18a, miR-34a, miR-106b, miR-183, miR-200a, miR-301a, miR-141, miR-182, miR-200b and miR-375, were measured in PCa (*n* = 31) and BPH (*n* = 31) patients. Compared to BPH, PCa samples showed significantly increased expression of miR-141, miR-182, miR-200b, and miR-375, which correlated with PSA levels. miR-200b levels showed concordance with Gleason score and among the miRNAs measured, ROC analysis indicated miR-200b as the best diagnostic biomarker (AUC 0.923, 95% CI 0.8618–0.9842) [167].

The levels of miR-21-5p, miR-141-3p, and miR-205-5p were proposed by Ghorbanmehr and colleagues for PCa diagnosis [168]. The study recruited PCa (*n* = 23) and BPH (*n* = 22) patients as well as healthy controls (*n* = 20). The levels of the 3 miRNAs measured in urine samples were found to increase in PCa and PBH compared to control and statistically segregated the 3 populations.

The study by Fredsøe and colleagues evidenced the usefulness of the miR-222-3p-miR-24-3p/miR-30c-5p ratio as a diagnostic tool for PCa [169]. The levels of 45 preselected miRNAs were measured in urine samples from patients with clinically localized PCa (*n* = 758) or BPH (*n* = 289) as well as in subjects undergoing transrectal ultrasound-guided biopsy (*n* = 233). Compared to BPH, PCa patients showed 21 downregulated and 8 upregulated miRNAs. The diagnostic model statistically segregated BPH from PCa patients (AUC 0.84) and predicted biopsy results better than PSA alone (AUC 0.66 and 0.527, respectively) for subjects with PSA lying in the grey area.

Urine samples were collected from patients with PCa (*n* = 10), BPH (*n* = 8) and healthy (*n* = 11) volunteers and the levels of 12 miRNAs measured. The analysis resulted in 8 miRNAs that combined in 6 ratios (miR-125b/miR-30e, miR-200/miR-30e, miR-205/miR-30e, miR-31/miR-30e, miR-660/miR-30e, and miR-19b/miR-92a) significantly separated the 3 populations. Although interesting, the study was performed in a short cohort (*n* = 8–11) and a large-scale validation study is mandatory [170].

Subjects with suspected PCa and undergoing prostate biopsy were enrolled in the study by Borkowetz and coworkers [171]. The study collected urine samples from 50 individuals, 26 of which diagnosed for PCa, and the levels of 12 miRNAs associated with PCa were measured. ROC analysis revealed the diagnostic power of miR-16 (AUC 0.744, *p* = 0.012; accuracy = 76%) and miR-195 (AUC 0.729, *p* = 0.017; accuracy = 70%), which increased following the combination with PSA density (AUC 0.801–0.849, *p* < 0.05; accuracy = 76–90%). Of note, miR-16 and miR-195 levels in combination with PSA density showed diagnostic potential (AUC 0.772–0.882, *p* < 0.05; accuracy = 74–85%) superior to PSA density alone (AUC 0.595, *p* = 0.524; accuracy = 68%) in patients with PSA levels lying in the grey area.

### 5.2. Prognostic miRNA Profiles

The serum levels of miR-20a and miR-26a were recently proposed for monitoring PCa patients after surgery. The study by Mohammadi Torbati and coworkers enrolled PCa patients (*n* = 20) and healthy subjects (*n* = 20) and the serum levels of the 2 miRNAs measured before and after prostatectomy of PCa patients. Comparing the expression levels of these miRNAs, the authors reported a downexpression of both miRNAs after surgery, with miR-20a showing the best statistic performance [172].

An interesting serum biomarker for monitoring PCa-derived bone metastasis is miR-218-5p. The study by Peng et al. collected both bone tissue and plasma samples from PCa patients suffering (*n* = 38) or not (*n* = 115) from bone-metastasis and the levels of miR-218-5p measured. The expression profile of bone and plasma samples showed concordance. Compared to nonmetastatic, reduced levels of the miRNA were evidenced in metastatic PCa patients (AUC 0.86, 95% confidence interval 0.80–0.92, *p* < 0.001). Of note, the low levels of miR-218-5p positively correlated with the poor prognosis of metastatic PCa subjects [173].

In another study, Zedan and colleagues proposed a prognostic miRNA profile for monitoring PCa following treatment [174]. Circulating miRNA signature based on plasma levels of miR-21, miR-93, miR-125b, and miR-221 was used for monitoring local or locally advanced PCa patients after medical intervention (radiotherapy, prostatectomy, ADT) in comparison with observation. The levels of miR-221 and miR-93 lowered following treatment and miR-93 expression significantly correlated with the Gleason score. The levels of miR-125b and miR-221 predicted risk assessment.

The expression of miR-424 and miR-572 was proposed by Suer and coworkers for the prediction of PCa progression [175]. In the study, recurrent (*n* = 20) and non-recurrent (*n* = 20) PCa patients were enrolled, and the serum levels of miRNAs were measured. The two groups showed a total of 682 deregulated miRNAs, among which the downregulation of miR-424 and the upregulation of miR-572 significantly correlated with recurrent PCa and predicted disease progression.

A miRNA profile including miR-141, miR-200a, and miR-375 was investigated by Cheng et al. as a prognostic tool for metastatic hormone-sensitive PCa [176]. Plasma samples from metastatic hormone-sensitive PCa patients treated with ADT in combination with cixutumumab or exposed to ADT alone were analyzed for miRNA expression. The patients were also analyzed for circulating tumor cells (CTCs) and PSA levels at baseline and after 28 weeks from treatment. CTCs and PSA showed concordance with miRNA levels in predicting therapy response. ROC analysis indicated that among the miRNAs considered, miR-375 showed the best performance with no significant differences compared to CTCs and PSA.

The expression of miR-223, miR-24, and miR-375 in combination with PSA levels was proposed by Liu et al. for the reclassification of AS patients [177]. Serum samples from AS patients were collected before potential reclassification and 9 miRNAs, previously found to associate with PCa progression, were measured. Statistical analysis, including logistic regression and ROC, applied on miRNA levels and clinical variables, identified 3 miRNAs scores, which predicted patient reclassification. The power of the ROC was impressively incremented by combining the 3 miRNAs score with PSA levels.

A novel liquid biopsy showing interesting prognostic power for mCRPC patients treated with enzalutamide was recently published by Benoist and coworkers [178]. Blood samples from 30 healthy subjects and 40 mCRPC patients were collected at baseline and 1, 3, and 6 months after the start of the treatment, and the levels of 4 mRNAs, 6 miRNAs, and 5 lncRNAs were measured. Compared to control individuals, the levels of miR-375, miR-3687, and N-acetylated alpha-linked acidic dipeptidase like 2 antisense RNA 2 (NAALADL2-AS2) lncRNA were significantly increased in mCRPC patients. A shorter time to progression-free survival was observed in patients with higher miR-375 and miR-3687 levels. Conversely, higher NAALADL2-AS2 expression is associated with a longer time to progression-free survival.

Patients under AS are periodically monitored by DRE, PSA tests, and biopsies. In case signs of tumor progression are recognized, patients are reclassified as high risk disease. Aimed at avoiding invasive medical practice, Zhao and colleagues proposed a combination of DNA methylation and miRNAs measured in urine samples of patients under AS [179]. The study collected post-DRE urine samples (*n* = 103) of subjects under AS and evaluated the DNA methylation of selected genes and 10 miRNA levels, previously observed to associate with AS reclassification. ROC and linear regression analysis identified miR-24, miR-30c and CRIP3 gene methylation that combined with PSA levels significantly predicted patient reclassification better than PSA alone.

### 5.3. Diagnostic and Prognostic miRNA Profiles

The expression of 5 miRNAs (miR-193a-3p, miR-9-3p, miR-335-5p, miR-330-3p, and miR-345-5p) was evaluated in the serum samples of patients affected by localized PCa, hormone-sensitive metastatic PCa, CRPC, and healthy individuals. The overexpression of miR-9-3p, miR-330-3p-3p, and miR-345-5p observed in PCa samples significantly separated PCa patients from controls. Despite the miRNA profiles were unable to segregate PCa groups, lower levels of miR-345-5p were recognized in patients treated with ADT. Although the investigation was performed in a short-cohort (*n* = 20–25), the study indicated miR-345-5p as a potential biomarker for PCa diagnosis and therapeutic response [180].

The levels of miR-182-5p and miR-375-3p were evaluated in prostate tissues and plasma samples of PCa patients (*n* = 252) and controls (*n* = 52). Increased levels of miR-182-5p were observed in both tissues (AUC 0.81, 95% CI: 0.725–0.892, *p* = 0.0001) and plasma (AUC 0.64, 95% CI: 0.561–0.709, *p* = 0.0021) of PCa patients compared to control subjects. Furthermore, a comparative evaluation of miRNA levels and clinical variables showed that miRNA expression predicted stage disease and metastasis. Specifically, miR-375-3p levels robustly identified patients that would suffer from metastasis at the time of diagnosis [181].

In the study by Kolluru and colleagues miR-301a was evaluated in serum and biopsies of PCa (*n* = 32, Gleason score 6–7) and BPH (*n* = 13) patients [182]. The results showed a miRNA overexpression in both tissue and serum samples of PCa compared to BPH patients. Moreover, the higher levels of miR-301a found in PCa in comparison to BPH biopsies correlated with the Gleason score.

Serum samples from subjects suffering from PCa (*n* = 809), from individuals with suspected PCa and negative biopsies (*n* = 241), from patients affected by other cancer types (*n* = 500), and from healthy controls (*n* = 41) were considered in a large-cohort study aimed at defining a diagnostic/prognostic miRNA signature. The candidate miRNAs for PCa were identified in a discovery set and the results were used to set up a diagnostic model in a training set. miRNA panel resulting from the discovery set included 18 miRNAs, among which the combination of miR-17-3p and miR-1185-2-3p resulted from the training set were used for the construction of the diagnostic model. Validation set (*n* = 484) indicated that the diagnostic model robustly identified PCa patients with the significant concordance with Gleason score and metastasis stage [183].

The expression levels of miR-17, miR-192, and miR-181a were proposed by Farran and colleagues for segregating aggressive and non-aggressive PCa [184]. Plasma samples (*n* = 114) belonging to the two PCa populations according to clinical variables were collected and analyzed for miRNA levels. The results, matched with clinical conditions of the patients, demonstrated that the panel significantly separated aggressive and non-aggressive PCa patients.

In the study by Zedan and coworkers, a circulating miRNA signature for metastatic PCa was proposed [185]. Tumor tissue and plasma samples were collected from metastatic and localized PCa patients as well as controls and miRNA expression analyzed. No perfect match was observed between miRNA levels in plasma and tissues. In plasma, miR-21, miR-125b, miR126, miR-141, and miR-375 were upregulated and let-7b reduced in metastatic PCa compared to controls. The expression of miR-93 showed concordance between tissues and plasma and significantly decreased in the blood following medical intervention on localized PCa.

The expression of miR-15a and miR-16-1 combined with PSA levels was also proposed for the diagnosis and prognosis of PCa patients [186]. The study by Zidan and coworkers recruited PCa (*n* = 70), BPH (*n* = 70), chronic prostatitis (CP, *n* = 30) subjects as well as controls (*n* = 70). Serum levels of miR-15a, miR-16-1, and PSA were measured. The serum levels of both miRNAs were decreased in PCa compared to BPH, CP, and controls and inversely correlated with the higher Gleason score, tumor stage, metastasis, and lymph node involvement. Moreover, the ROC analysis demonstrated that the combination of miR-15a, miR-16-1, and PSA was better than PSA alone in PCa diagnosis.

The expression levels of miR-1825, miR-484, miR-205, miR-141, and let-7b in the serum of PCa (*n* = 72) patients and healthy (*n* = 34) subjects were considered in the study by Guo et al. [187]. The 5 miRNAs were significantly upregulated in the serum of PCa compared to control subjects. Since the levels of miR-205 and miR-1825 correlated with bone metastasis and lymph node involvement, and that of let-7b predicted ADT resistance, the signature was proposed as a powerful tool for PCa diagnosis, prognosis as well as drug therapy response.

The amounts of miR15a, miR-126, miR-192, and miR-377 were proposed by Al-Kafaji et al. as a diagnostic tool to distinguish localized PCa from BPH [188]. The study collected blood samples from PCa (*n* = 35), BPH (*n* = 35) patients, and healthy (*n* = 30) subjects, and miRNA levels were measured. A reduced expression of miR15a, miR-126, miR-192, and miR-377 was revealed in PCa compared to BPH patients. Moreover, the comparison of miRNA levels with the clinical variables (e.g., tumor stage, Gleason score, PSA levels), which allow the categorization in low- and high-risk groups, showed a significant correlation between the lower levels of the 4 miRNAs and the high-risk group.

## 6. Conclusions

The field of miRNAs as therapeutics and biomarkers for PCa is still an attractive and growing area of research.

Despite the success reached by miRNAs as laboratory tools, and the numerous studies published thus far, the use of nucleic acids as PCa-selective drugs showed inconclusive results. The clinical trials containing miRNAs revealed unacceptable toxic effects often requiring the withdrawn of the studies.

In this review, we also emphasize some of the needs still unmet in relation to the detection of miRNAs into the biofluids and to their usefulness in unambiguously define miRNA signatures in PCa. The identification of the best patient specimen to analyze is crucial for a reliable liquid biopsy. miRNA profiles of peripheral blood contain miRNAs released from the blood cells and healthy tissues. Conversely, urine miRNAs are supposed to be less contaminated than those released from healthy organs, even if unstable because of the extreme environmental conditions. This problem could be bypassed by measuring miRNAs packaged into the EVs. However, signatures focused on EVs miRNAs imply the loss of numerous unpackaged nucleic acids likely carrying important cancer-related information. The tumor cell specificity of the liquid biopsy also depends on the tumor microenvironment. Cells composing the tumor microenvironment (cancer-associated fibroblasts, endothelial cells, and blood cells) interact with cancer cells and with each other by releasing miRNA “messengers”, which invade biologic fluids and interfere with the tumor specificity of the screening test. These features likely reflect the variability observed for some miRNAs in different studies (e.g., upregulated in some cases and downregulated in some others) and would account for the discrepancy showed between miRNA profiles from prostate tissues and liquid biopsies. Finally, the investigations are often performed in short-cohorts of patients, and validation studies in large-cohorts are lacking.

For the future, we envision that the development of innovative biocompatible and biodegradable nanomaterials for nucleic acid delivery will continue the booming wave. As far as the use of miRNAs as PCa specific biomarkers are concerned, additional efforts aimed at solving the numerous analytical biases are mandatory to achieve reliable data. Although the numerous flaws, PSA remains the best biomarker available for PCa management. The combination of miRNA profiles and PSA showed interesting results, particularly in patients with ambiguous PSA levels. To date, miRNA translation into clinical practice for PCa appears still uncertain.

## Figures and Tables

**Table 1:** miRNA delivery approaches for prostate cancer treatment.

Delivery Strategy	Mechanism of Action
**Onco-miRNA silencing**	
ASO	Single-stranded RNAs annealing with miRNAs
miRNA sponges	RNAs containing multiple tandem binding sites
Small molecules	Chemicals that recognize peculiar structures of a specific miRNA and interfere with its biogenesis or maturation
CRISPR/Cas9	Genome editing of miRNA-containing gene allowing suppression of miRNA expression
**Tumor suppressor miRNA replacement**	
miRNA mimics (ago-miRNAs)	Chemically modified double-stranded RNA molecules designed to mimic endogenous miRNAs
Viral vectors	Viral vectors encoding for miRNAs that improve miRNAs expression
Small molecules	Chemicals that improve the endogenous expression of miRNAs by epigenetic changes within the gene structure

**Table 2:** miRNA profiles endowed with diagnostic and prognostic potential in prostate cancer ^a^.

miRNA	Modulation (PCa vs. Controls)	Patient Specimen
**Diagnostic miRNA profiles**		
miR-1246	Increased	Serum
miR-150-5p	Reduced	Plasma
miR-141-3p, miR-21, miR-375	Increased	Serum
miR-141	Increased	Plasma
miR-98-5p, miR-152-3p, miR-326, miR-4289	Increased	plasma
miR-4286, miR-27a-3p, miR-29b-3p	Reduced	Serum
miR-10a-5p, miR-29b-3p	Increased	plasma
miR-21, miR-141, miR-18a, miR-221	Increased	plasma
miR-494	Increased	Serum
miR-103a-3p, let-7a-5p	Increased	plasma
miR-324	Increased	Serum
miR-375-miR-33a-5p/miR-16-5p-miR-409-3p	n.d	plasma
miR-125a-5p/miR-141-5p	reduced/increased	plasma
miR-374-5p	Increased	Serum
miR-141, miR-182, miR-200b, miR-375	Increased	Serum
miR-21-5p, miR-141-3p, miR-205-5p	Increased	Urine
miR-222-3p-miR-24-3p/miR-30c-5p	n.d	Urine
miR-125b/miR-30e, miR-200/miR-30e miR-205/miR-30e, miR-31/miR-30e miR-660/miR-30e, miR-19b/miR-30e	n.d	Urine
miR-195, miR-16	Increased	Urine
**Prognostic miRNA profiles**		
miR-20a, miR-26a	Reduced	Serum
miR-218-5p	Reduced	Serum
miR-93, miR-221, miR-126b	Reduced	plasma
miR-424, miR-572	reduced/increased	Serum
miR-141, miR-200a, miR-375	Increased	plasma
miR-223, miR-24, miR-375	n.d.	Serum
miR-375, miR-3687	Increased	Serum
miR-24, miR-30c	Increased	urine
**Diagnostic and prognostic miRNA profiles**		
miR-9-3p, miR-330-3p-3p, miR-345-5p	Reduced	Serum
miR-182-5p, miR-375-3p	Increased	plasma
miR-301a	Increased	Serum
miR-17-3p, miR-1185-2-3p	Increased	Serum
miR-17, miR-192, miR181a	Increased	plasma
miRNA-21, miR-125b, miR-126, miR-141, miR-375, let-7b	Increased	plasma
miR-15a, miR-16-1	Reduced	Serum
miR-1825, miR-205, let-7b	Increased	Serum
miR-15a, miR-126, miR-192, miR-377	Reduced	Serum

^a^ miRNA signatures published during the last 3 years are summarized. n.d. not reported.

## Data Availability

Not applicable.
